# Eastern filbert blight resistant *Corylus avellana* identified from 20 years of germplasm introduction and evaluation at Rutgers University, New Jersey, USA

**DOI:** 10.3389/fpls.2024.1502392

**Published:** 2024-12-17

**Authors:** Daniel C. Jacobs, Ronald S. Revord, John M. Capik, Thomas J. Molnar

**Affiliations:** ^1^ Department of Plant Biology, Foran Hall, Rutgers University, New Brunswick, NJ, United States; ^2^ Center for Agroforestry, School of Natural Resources, University of Missouri, Columbia, MO, United States

**Keywords:** hazelnut, germplasm curation, disease screening, breakdown, “Gasaway”

## Abstract

The stem canker disease eastern filbert blight (EFB), caused by *Anisogramma anomala*, is a major impediment of European hazelnut (*Corylus avellana*) production in the United States. While most European hazelnut cultivars are highly susceptible to the pathogen, which remains confined to North America, EFB resistant and tolerant genotypes occur in the gene pool at low frequency. At Rutgers University, New Brunswick, NJ, USA, 5,226 trees were grown from open pollinated seeds collected from Russia, Crimea, Poland, Turkey, Estonia, Latvia, Lithuania, Moldova, Azerbaijan, Italy, and the Republic of Georgia between 2002 to 2010. The trees were field planted, exposed to *A. anomala* under high pathogen pressure, and evaluated for disease response 5-6 years after their establishment. At this point, around four percent were found to be EFB resistant totaling 216 accessions that spanned a wide diversity of seedlots from most countries and regions. However, recent observations show many of these once-resistant selections have since succumbed to EFB. In this study, the long-term disease response of this germplasm was evaluated to identify trees remaining resistant and tolerant and document changes in EFB response over time in relation to their origin. All trees were rated for presence of EFB according to a scale of 0 to 5 where 0 = no EFB and 5 = all stems have cankers. Data were assembled from three sets: first reports from 5-6 years after each planting year, a reassessment in 2017, and a final evaluation in January 2024. Overall, the results showed a significant reduction in resistant individuals from the original reports. By 2017, the population of 216 trees was reduced to 154 and by 2024 it decreased further to 91. Notably, this shift from resistant to susceptible phenotype was severe and abrupt and patterns were observed within related seed lots. These patterns were also apparent in trees where their resistance (*R*) genes were mapped. Specifically, all selections with *R* genes mapped to linkage group (LG) 6 now expressed severe EFB, while those with *R* genes mapped to LG 2 or 7 remained free of disease. These results strongly suggest pathogenic variation present over time played a role in the breakdown of resistance. Fortunately, despite loss of some of the germplasm, a wide variety of trees spanning most collection origins still remain free of EFB. These 91 trees from 56 distinct seedlots originating from 7 countries are formally documented in this manuscript to facilitate their long-term preservation, continued evaluation and sharing, and to increase global awareness of this valuable genetic resource for future research and breeding.

## Introduction

The *Corylus* genus comprises 13 species native to a wide area of the northern hemisphere; all are monoecious, wind pollinated, self-incompatible, and have edible nuts. Of the genus, the European hazelnut (*C. avellana*) is the main species grown commercially for nut production (Botta et al., 2019; Molnar, 2011). While wild *C. avellana* is commonly found throughout much of Europe, spanning north of Moscow, Russia, to Scandinavia and south into the Caucasus region and parts of western Asia, commercial cultivation exists primarily in locations near large bodies of water with mild, Mediterranean-like climates. World production is based on a small number of clonally propagated cultivars selected from local seedling populations, many whose origins have been lost in antiquity (Botta et al., 2019; Mehlenbacher and Molnar, 2021). Major producing countries include Turkey with about 65% of the world’s crop, followed by Italy (~12-15%), Azerbaijan (~5%), the United States (~5%), and the Republic of Georgia (~3%), with additional notable production in Chile, China, and France (Food and Agricultural Organization of the United Nations, 2021).

Eastern filbert blight (EFB), a perennial stem canker disease caused by *Anisogramma anomala*, is the primary limiting factor for hazelnut production in North America (Johnson and Pinkerton, 2002; Thompson et al., 1996). *Anisogramma anomala* is an obligate biotrophic ascomycete in the order Diaporthales and is specific to only *Corylus* spp. It reproduces and spreads by ascospores, which infect actively growing shoot tips during wet conditions in the spring, and by perennial canker expansion within infected trees (Johnson et al., 1994; Pinkerton et al., 1998a, b). The pathogen is endemic to a wide area of the eastern U.S. and southern Canada where it is found associated with its native host *Corylus americana*, the wild American hazelnut. Genetic fingerprinting studies have shown it to be highly genetically diverse across its native range and with an extremely large genome for a fungus (>360Mb) (Cai et al., 2013; Muehlbauer et al., 2019; Tobia et al., 2024; Cohen et al., 2024). The occurrence of EFB on *C. americana* is generally mild, mostly exhibiting as occasional stem cankers that are small in size (<25 cm) and have little impact on plant health (Capik and Molnar, 2012; Revord et al., 2020). In contrast, the European hazelnut, *C. avellana*, the species cultivated for its nuts, is in general highly susceptible to EFB. The disease is expressed as devastating large stem cankers, sometimes > 1.0 m long, that disrupt vascular tissues and can lead to tree death just a few years after exposure (Mehlenbacher, 1991; Pinkerton et al., 1993; Capik and Molnar, 2012; Jacobs et al., 2024).

Native populations of *C. americana* span much of the eastern United States and act as a perennial reservoir of inoculum to infect any *C. avellana* planted across its range. As such, this disease has thwarted attempts at hazelnut production in this region since colonial times (Thompson et al., 1996). It is important to note that *A. anomala* remains confined to North America. Strict quarantine measures are in place to help prevent its spread to other regions of the world. The European and Mediterranean Plant Protection Organization (EPPO) considers this pathogen a level A1 quarantine pest (EPPO, 2024).

Currently, ~99% of U.S. commercial hazelnut production occurs in the Willamette Valley of Oregon. Historically, the industry thrived for nearly 100 years outside the native range of *A. anomala* which was originally confined East of the Rocky Mountains. The threat of EFB was recognized early and the movement of *Corylus* from eastern regions into the western U.S. was prohibited to prevent its spread (Fuller, 1908; Barss, 1930), which was effective for many decades. However, the industry was challenged with the inadvertent introduction of *A. anomala* in the 1960s (Davison and Davidson, 1973). Lacking control measures at the time, EFB devastated orchards from the point of its introduction in southwest Washington then south into the Willamette Valley; many orchards were lost, and the industry was reduced in size (Gottwald and Cameron, 1980). Production was based mostly on the *C. avellana* cultivar Barcelona and its pollinizer Daviana (Mehlenbacher and Miller, 1989), which provided a relatively uniform susceptible host population. Fortunately, the main cultivar Barcelona expressed a small level of tolerance to EFB (continuing to produce crops while infected), allowing orchards to be maintained with scouting and pruning of infected limbs, coupled with fungicide applications (Johnson et al., 1996). However, these management tactics were not always fully effective and significantly increased costs of production for this otherwise low-input crop (Julian et al., 2008).

Fortunately, genetic resistance to EFB in *C. avellana* was identified in ‘Gasaway’; an obsolete, late-blooming pollinizer with low yields of oblong nuts (Cameron, 1976; Mehlenbacher et al., 1991). Since its discovery, ‘Gasaway’ has been utilized extensively at Oregon State University (OSU), Corvallis, OR, in breeding efforts to develop EFB resistant cultivars with improved nut quality. These efforts led to the release of ‘Jefferson’, ‘Yamhill’, and ‘McDonald’, among others, and several dedicated pollinizers, all of which carry the dominant EFB resistance allele from ‘Gasaway’ (Mehlenbacher et al., 2023). These new cultivars have been widely accepted by growers and have facilitated a rapid expansion of the hazelnut industry in the Willamette Valley from 12,000 ha in 2009 to over 35,000 ha today (Mehlenbacher, 2018; Mehlenbacher et al., 2023).

The discovery of ‘Gasaway’ suggested that other sources of resistance could be found within *C. avellana*, which, coupled with concerns over the durability of using only one single resistance (*R*) gene, led to wide germplasm screening efforts. Several hundred *C. avellana* and interspecific hybrid accessions housed in the OSU germplasm collection and the USDA National Clonal Germplasm Repository (Corvallis, OR) were challenged with *A. anomala* (Mehlenbacher, 2018). From this work, new *C. avellana* accessions resistant or highly tolerant to EFB were identified including ‘Ratoli’ from Spain, OSU 759.010 from the Republic of Georgia, OSU 495.072 from southern Russia, and 408.040 from Minnesota (U.S.), among others (Coyne et al., 1998; Chen et al., 2007; Lunde et al., 2000; Sathuvalli et al., 2009; Sathuvalli et al., 2011a, b; Mehlenbacher et al., 2023; Mehlenbacher and Molnar, 2021).

New seed-derived germplasm was also evaluated. Since hazelnuts are wind-pollinated and self-incompatible, significant genetic diversity can be captured through collecting open pollinated (OP) seeds from locally derived clonal cultivars grown in regions where wild trees are often the pollen donors (Campa et al., 2011; Mehlenbacher and Molnar, 2021). At Rutgers from 2002-2010, OP hazelnut seeds were obtained from Armenia, Azerbaijan, Crimea, Estonia, the Republic of Georgia, Italy, Latvia, Lithuania, Moldova, Poland, Russia, and Turkey. Their acquisition spanned multiple collection trips organized by T. Molnar, S. Mehlenbacher, D. Zaurov, and/or M. Pisetta as summarized in Molnar et al. (2018) and Molnar (2022). Specifically, the collections from Russia and Crimea are detailed in Molnar et al. (2007) and Capik et al. (2013), Poland in Capik et al. (2013), and the Republic of Georgian in Molnar and Pisetta (2009) and Leadbetter et al. (2015). Smaller collection efforts also yielded plant materials that are included in Muehlbauer et al. (2014) and Lombardoni et al. (2024) and described in more detail subsequently.

Collection trips were timed to coincide with nut harvest per country (August-September) and seeds in the form of fresh, in-shell nuts were obtained from a wide variety of markets, bazaars, roadside stands, backyard gardens, farms, research institutes, and botanical gardens. They were organized with local scientists and their research institutes who facilitated travel, permitting, and phytosanitary certifications for export to the United States. In many cases, seeds were shared between Rutgers and OSU for evaluation at both locations (note that only Rutgers evaluated materials are described in this manuscript).

In total, 5,226 trees were field planted and subjected to the high and genetically diverse *A. anomala* pressure (see Tobia et al., 2024) present at the Rutgers research farms. A vast majority of the trees were highly susceptible and died from severe EFB within several years after planting; however, 216 trees (~4%) remained resistant or highly tolerant (few cankers per tree, and/or smaller cankers with reduced impact) following evaluations completed 5-6 years after their respective planting dates (**Table 1**). These results were especially promising as the new trees greatly increased the pool of EFB resistance sources available at that time. Further, subsequent fingerprinting studies showed high genetic diversity was present among these trees, with results indicating the presence of at least 14 distinct genetic groups and subgroups amongst the selections; it also supported that their use in breeding for resistance to EFB would not necessarily lead to a narrowing of genetic diversity (Muehlbauer et al., 2014; Lombardoni et al., 2024). Recent genetic mapping studies have identified the presence and location of major *R*-genes carried by some of these selections, with *R*-genes localized to 3 of the 11 linkage groups (LGs) on the *C. avellana* genetic map (Mehlenbacher et al., 2006, 2023). Of the nine selections studied, six had *R*-genes mapped to LG6 (the same region as the ‘Gasaway’ resistance allele), two to LG7, and one to LG2 (Honig et al., 2019; Şekerli et al., 2021; Komaei Koma et al., 2021; Mehlenbacher et al., 2023). Additionally, several trees exhibiting a high level of tolerance (reduced disease severity) resembling quantitative resistance (QR) to EFB were also identified from the original screening efforts, adding to the diversity of trees available for resistance breeding (Capik et al., 2013; Leadbetter et al., 2015).

**Table 1:** Summary of Rutgers University *Corylus avellana* germplasm collection trips including numbers of seedlots and subsequent trees evaluated in the field.

Collection origin^a^	Year Collected	No. of Distinct Seedlots	No. of Trees in Field^b^	Seedlots with Resistant Trees (2017)	No. of Trees Free of EFB (2017)	Seedlots with Resistant Trees (2024)	No. of Trees Free of EFB (2024)	Collectors	References
Russia (Krasnodar, Maykop, Holmskij, Sochi)	2002	20	981	10	29	7	12	T. Molnar, D. Zaurov, S. Mehlenbacher	Molnar et al., 2007; Capik et al., 2013
Crimea (Yalta, Simferopol)	2002	12	304	6	9	4	6	T. Molnar, D. Zaurov, S. Mehlenbacher	Molnar et al., 2007; Capik et al., 2013
Russia (Sochi)	2004	23	749	9	36	7	9	D. Zaurov	Molnar et al., 2007; Capik et al., 2013
Turkey (Giresun)	2004	50	509	8	9	3	5	S. Mehlenbacher	NA
Lithuania	2005	6	40	1	1	0	0	T. Molnar, D. Zaurov	NA
Latvia	2005	5	304	2	5	1	2	T. Molnar, D. Zaurov	NA
Poland (Warsaw, Skiemiewice, Konskowli)	2006	14	415	3	6	2	5	T. Molnar, D. Zaurov, M. Wojciechowska, G. Hodun	Capik et al., 2013
Estonia (Tartu and Polli)	2007	4	203	3	3	3	3	K. Kask, T. Paal	NA
Moldova	2007	4	144	2	5	0	0	T. Molnar, D. Zaurov	NA
Russia (Moscow)	2008	7	61	1	2	1	1	D. Zaurov	NA
Republic of Georgia	2009	46	1372	29	48	27	47	M. Pisetta, N. Mirotadze	Leadbetter et al., 2015
Russia (southern)	2009	1	2	1	1	1	1	D. Zaurov	NA
Azjerbaijan	2009	2	36	0	0	0	0	M. Pisetta, N. Mirotadze	Leadbetter et al., 2015
Italy (Napoli, Caserta)	2010	4	106	0	0	0	0	M. Pisetta	NA
	Totals	198	5226	75	154	56	91		

The numbers of seedlots and trees exhibiting resistance and high tolerance to eastern filbert blight (EFB) in 2017 and 2024 are also shown.

^a^Collection origin includes country of origin followed by (region or city) where applicable.

^b^Number of individual seedling trees that successfully germinated and were planted at Rutgers University. NA, Not Applicable.

However, over the next decade, changes in disease response became evident for some hazelnut cultivars and selections in the Rutgers holdings that could not be attributed to escape from *A. anomala* exposure. Most notably, cultivars and selections protected by the ‘Gasaway’ *R*-gene, which originally remained free of EFB in the plots at Rutgers, began to develop small cankers (Molnar et al., 2010b; Capik and Molnar, 2012). Concurrent controlled inoculation studies indicated that pathogenic differences between populations of *A. anomala* likely played a role in this shift in disease expression (Molnar et al., 2010a). While the early field infections were generally mild and had little impact on overall tree health (Capik and Molnar, 2012; Muehlbauer et al., 2018), starting in 2017 ‘Gasaway’ *R*-gene protected cultivars such as ‘Jefferson’ and ‘Yamhill’ began to fully succumb to EFB. These trees exhibited susceptible phenotypes with large cankers that lead to tree death, which was in drastic contrast to their response several years earlier. This dramatic change in disease response reflects the presence of a strain or population of *A. anomala* able to overcome the ‘Gasaway’ *R*-gene (Jacobs et al., 2024; Dunlevy et al., 2019; Mehlenbacher et al., 2023).

In a similar manner to trees protected by the ‘Gasaway’ source of resistance, starting around 2017 a proportion of the EFB “resistant” trees from the Rutgers’ *C. avellana* seed introductions also began to show dramatic changes in EFB response. Many trees that held up to high EFB pressure, in some cases for more than a decade, were now succumbing to disease. Due to these changes in disease development, the current EFB response status of this large body of germplasm is not reflected in the available published literature and an update is warranted to support the future use of the plant materials in resistance breeding and other research efforts. In this study, an examination of the total collection, representing all collection years and planting dates, was completed. Present-day longer-term EFB response was reported and compared to past records. Trends observed among seed lots and seed origins were also examined to help elucidate drivers of the breakdown of resistance. Ultimately, these efforts are reported herein to highlight the remaining resistant and tolerant accessions to facilitate their future utilization by the hazelnut scientific community.

## Materials and method

### Plant materials

**Figure 1:**
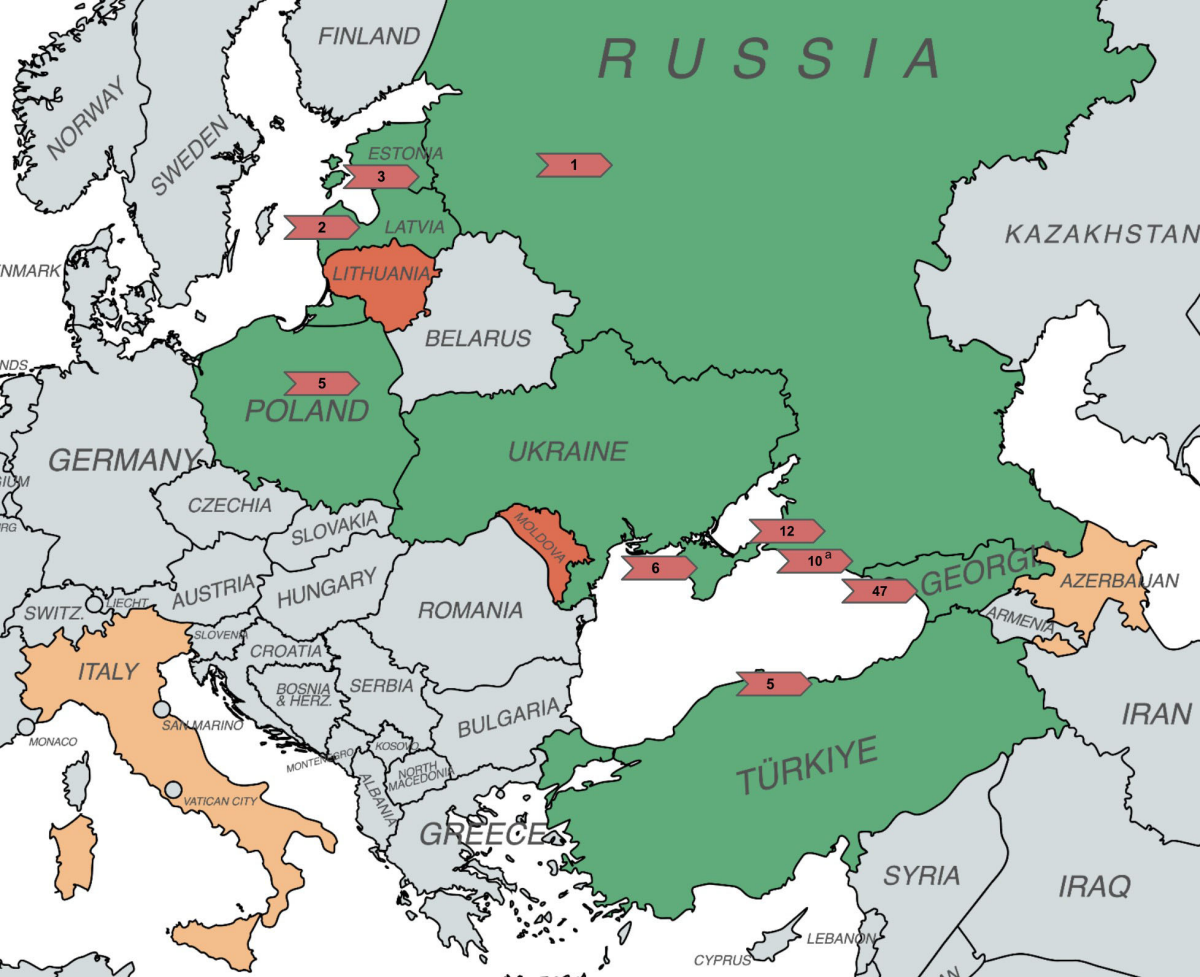
Map detailing *Corylus avellana* germplasm collection locations included in this study. Red arrows denote regions where the eastern filbert blight resistant trees in this study originate from, including the number of individuals remaining from these specific regions. Countries from which resistant trees were identified and persist at Rutgers University are shaded in green, countries from which resistant trees were identified and no longer persist at Rutgers University are dark orange, and countries from which no resistant trees were recovered are light orange. (Map generated using MapChart, https://www.mapchart.net/europe.html). **^a^** Eastern filbert blight resistant accession HF1BR21P182 (Russian Unknown 1) originating from an unknown location in southern Russia is included in the count of trees from the Sochi region where it is suspected to originate from.

The direct disease evaluation component of this study completed in 2024 comprises 154 individual trees surviving from the large OP seedling populations described previously. These specifically include: 67 trees from Russia; 49 from the Republic of Georgia; 9 from Crimea; 9 from Turkey; 6 from Poland; 5 from Latvia; 5 from Moldova; 3 from Estonia; and 1 from Lithuania (**Table 1**; **Figure 1**). Note that this study also references published manuscripts and unpublished Rutgers University breeding records of additional trees once deemed resistant but that are no longer existing in the field collection (due to succumbing to EFB).

Originally, seeds were germinated and trees planted in the field in September or October the year after their collection, which spanned from 2002 to 2010. They were organized in blocks by seedlot at a spacing of 0.45 or 0.91 m within rows and 3.66 m between rows depending on the year and space available. The evaluation plots were established in New Jersey at either the Rutgers Fruit Research and Extension Center, Cream Ridge; the Horticulture Research Farm 3, East Brunswick; or the Horticulture Research Farm 1, New Brunswick. Irrigation, fertilization, and weed control were performed in the plots as needed. No applications of fungicide or insecticides were performed. Dead trees were documented and then removed from plots annually to assist in plot management.

To place the timeline in perspective, note that the oldest trees in this study have been exposed to *A. anomala* for 21 years (field planted in 2003) and the youngest for 14 years (field planted in 2010). Those trees alive in 2024 have remained in place in their original planting positions and were left to develop into their natural multi-stemmed form with no pruning. These trees exist across multiple locations and fields at the respective research farms. They were each assigned field location identification codes denoting the farm at which they were planted, along with their relative location in the field (row and tree number). Trees planted at Cream Ridge Fruit Research and Extension center are denoted by the codes CRR, CRT, and CRX, and those at the Horticulture Research Farms as HF1, HF3, H4A, and H4B. Accession names and other aliases have also been assigned to several trees in previous publications and are included in **Table 2**.

**Table 2:** The final set of 91 *Corylus avellana* accessions derived from open pollinated seed collection efforts spanning 2002 to 2010 that remain resistant or highly tolerant to eastern filbert blight caused by *Anisogramma anomala* at Rutgers University in New Jersey.

Accession Code^a^	Other Names/Aliases^b^	Seedlot Code	City, Country Origin	Parent Seed Source Information^c^ and Footnotes for Related Citations	Year Planted	Genetic Group^d^
CRXR13P108	Kudashovski’ OP #13	RUS-2	Sochi, Russia	‘Kudashovski’ open pollinated (OP), Inst of Flori. and Subtrop. Cultures^fgh^	2003	Mixed Group
CRXR14P34	Sochi Market 2 #1	RUS-4	Sochi, Russia	Sochi Market #2^fgh^	2003	Black Sea Group
CRXR14P42	Sochi Market 2 #2	RUS-4	Sochi, Russia	Sochi Market #2^fgh^	2003	Black Sea Group
CRXR14P44	Sochi Market 2 #3	RUS-4	Sochi, Russia	Sochi Market #2^fgh^	2003	Black Sea Group
CRXR14P47	Sochi Market 2 #4	RUS-4	Sochi, Russia	Sochi Market #2 ^fgh^	2003	Black Sea Group
CRXR14P97	Sochi Market 4 #1	RUS-6	Sochi, Russia	Sochi Market #4 ^fgh^	2003	Black Sea Group
CRXR15P07	Kudashovshi’ OP #20	RUS-2	Sochi, Russia	‘Kudashovski’ OP, Inst. of Flori. and Subtrop. Cultures ^fgh^	2003	Wild *C. avellana* group
CRXR15P50	Holmskij Market 3 #2	RUS-11	Maykop, Russia	Holmskij Market #3 ^fgh^	2003	Black Sea Group
CRXR15P59	Holmskij Market 3 #3	RUS-11	Holmskij, Russia	Holmskij Market #3 ^fgh^	2003	Black Sea Group
CRXR16P57^e^	Nikita Botanical Garden 1 #1	RUS-28	Yalta, Crimea	Nikita Botanical Garden #1^fgh^	2003	Black Sea Group
CRXR17P48	Maykop VIR #1	RUS-15	Maykop, Russia	OP seed mix, Vavilov Research Inst. of Plant Industry (VIR)^fgh^	2003	Wild *C. avellana* group
CRXR19P02	Simpferopol Market 5 #1	RUS-26	Simferopol, Crimea	Simferopol Roadside Market #5^fgh^	2003	No Group
H3R03P12	‘Badem’ OP #3	RUS 16	Krasnodar, Russia	‘Badem’ OP #3, Inst. Orchard and Wine Production^fghi^	2003	Black Sea group, Georgian 2
H3R07P25	Holmskij Market 4 #2	RUS 12	Holmskij, Russia	Holmskij Market 4^fghij^	2003	Black Sea 1, Black Sea
H3R10P88	Nikita Botanical Garden 1 #3	RUS 28	Yalta, Crimea	Nikita Botanical Gardens #1^fgh^	2003	Black Sea 2
H3R12P58	Simferopol Market 2 #2	RUS 23	Simpferopol, Crimea	Simferopol roadside market #2^fghk^	2003	Wild *C. avellana*
H3R12P62	Simferopol Market 2 #3	RUS 23	Simferopol, Crimea	Simferopol roadside market #3^fghl^	2003	Wild *C. avellana*
H3R14P26	Simferopol Market 1B #1	RUS 22	Simferopol, Crimea	Simferopol roadside market #1B^fghi^	2003	Black Sea Group, Georgian 4
CRRR01P116	B-X-3 OP #1	04041 R	Sochi, Russia	B-X-3 OP^ghi^	2005	Black Sea Group, Georgian 2
CRRR02P41	Rimski OP #2	04040 R	Sochi, Russia	‘Rimski’ OP^ghi^	2005	Spanish-Italian Group, Georgian 2
CRRR03P11	Sochi Unknown 3	04034 R	Sochi, Russia	Unknown seedling OP^gi^	2005	Georgian 2
CRRR04P19	‘Moskovskii Rubin’ OP #3	04030 R	Sochi, Russia	‘Moskovskii Rubin’ OP, Inst of Flori. and Subtrop. Cultures^gh^	2005	Moscow Group
CRRR04P107	Kavkas OP #1	04028 R	Sochi, Russia	‘Kavkas’ OP^gh^	2005	Black Sea Group
CRRR04P116	Kavkas OP #2	04028 R	Sochi, Russia	‘Kavkas’ x OP^gh^	2005	Black Sea Group
CRRR05P32	Sochi Unknown 2	04026 R	Sochi, Russia	Unknown Seed Mixture^gi^	2005	Georgian 1
CRRR06P50	President OP #3	04022 R	Sochi, Russia	President’ OP^gh^	2005	Black Sea Group
CRRR06P53	President OP #4	04022 R	Sochi, Russia	President’ x OP^gh^	2005	Black Sea Group
CRTR02P03	Giresun 233 1	OSU-04131	Giresun, Turkey	Giresun 233 Hazelnut Research Inst.^i^	2006	Black Sea Group 2, Admixed
CRTR02P13	NA	OSU-04102	Giresun, Turkey	Giresun 109 Hazelnut Research Inst.^i^	2006	Black Sea Group 2
CRTR02P138	Giresun 112 2	OSU-04104	Giresun, Turkey	Giresun 115 Hazelnut Research Inst.^i^	2006	Black Sea Group 2, Turkish
CRTR04P14	Giresun 286 1	OSU-04104	Giresun, Turkey	Giresun 286 Hazelnut Research Inst.^i^	2006	Black Sea Group 2, Turkish
CRTR04P124	Giresun 194 1	OSU-04104	Giresun, Turkey	Giresun 194 Hazelnut Research Inst.^i^	2006	Black Sea Group 2, A dmixed
CRTR06P95^e^	Riga Market 3 OP 1	Riga 3	Riga, Latvia	Riga Roadside Market 2^i^	2006	Wild *C. avellana*, Central European 1
CRTR06P100^e^	Riga Market 3 OP 2	Riga 3	Riga, Latvia	Riga Roadside Market 2^i^	2006	Wild *C. avellana*, Central European 1
H4AR20P88	Warsaw Market 4 #1	06080 P	Warsaw, Poland	Warsaw Market^gh^	2007	Central European 2
H4AR21P03	Warsaw Mix #1	06085 P	Warsaw, Poland	Unknown seed mixture, market^gh^	2007	Central European 2
H4AR21P05	Warsaw Mix #2	06085 P	Warsaw, Poland	Unknown seed mixture, market^gh^	2007	Central European 2
H4AR21P27^e^	NA	06085 P	Warsaw, Poland	Unknown seed mixture, market^g^	2007	NA
H4AR21P43	Warsaw Mix #3	06085 P	Warsaw, Poland	Unknown seed mixture, market^gh^	2007	Central European
H4BR17P67	NA	Est 1	Tartu, Estonia	T. Paal collection	2008	NA
H4BR22P55	Tartu Home Garden 1	Est 2	Tartu, Estonia	T. Paal collection^i^	2008	Georgian 1
H4BR22P108	NA	Est 3	Tartu, Estonia	T. Paal collection	2008	NA
H4BR21P137	Dacha 4	08561	Moscow, Russia	Dacha 4, Mamonova collection	2009	NA
HF1BR21P28	NA	09634	Kakheti, Georgia	Chkhivistava OP; Private Garden^m^	2010	NA
HF1BR21P163	Georgia Adjara 2	09612	Adjara, Georgia	Gulshishvela OP; roadside vendor^im^	2010	Georgian 3
HF1BR21P182	Russian Unknown 1	09675	Russia (southern)	unknown seed source from David Zaurov^i^	2010	Central European 1
HF1BR21P192	Georgia Guria 1	09620	Guria, Georgia	Kharistvala OP; market^im^	2010	Georgian 4
HF1BR23P14	Georgia Guria 2	09619	Guria, Georgia	Cincia OP; market^im^	2010	Georgian 3
HF1BR23P16	NA	09619	Guria, Georgia	Cincia OP; market^m^	2010	NA
HF1BR23P70^e^	Georgia Guria 3	09624	Guria, Georgia	Shvelis Kura OP; private garden^im^	2010	Georgian 2
HF1BR26P21	Georgia Adjara 4	09611	Adjara, Georgia	Berzula (Anakliuri) OP; roadside vendor^im^	2010	Georgian 1
HF1BR26P37	Georgia Adjara 5	09611	Adjara, Georgia	Berzula (Anakliuri) OP; roadside vendor^im^	2010	Georgian 1
HF1BR26P77	Georgia Kakheti 3	09637	Kakheti, Georgia	Mekutkasheni OP; private garden^im^	2010	Georgian 4
HF1BR26P138	Georgia Kakheti 4	09647	Kakheti, Georgia	Hybrids OP; private garden^im^	2010	Georgian 2
HF1BR26P140	Georgia Abkhazia 1	09616	Abkhazia, Georgia	Anakliuri OP; private garden^im^	2010	Georgian 4
HF1BR26P151	Georgia Kakheti 5	09648	Kakheti, Georgia	Pshauri 3 OP; private garden^im^	2010	Georgian 2
HF1BR26P162	Georgia Kakheti 7	09648	Kakheti, Georgia	Pshauri 3 OP; private garden^im^	2010	Georgian 2
HF1BR26P163	Georgia Kakheti 8	09648	Kakheti, Georgia	Pshauri 3 OP; private garden^im^	2010	Georgian 2
HF1BR26P167	NA	09638	Kakheti, Georgia	Pshauri OP; private garden^m^	2010	NA
HF1BR26P169^e^	NA	09638	Kakheti, Georgia	Pshauri OP; private garden^m^	2010	NA
HF1BR28P63	Georgia Guria 4	09620	Guria, Georgia	Kharistvala OP; market^im^	2010	Georgian 4
HF1BR28P68	Georgia Guria 5	09620	Guria, Georgia	Kharistvala OP; market^im^	2010	Georgian 5
HF1BR28P199	Georgia Adjara 6	09618	Samegrelo, Georgia	Giresum OP; market^im^	2010	Turkish
HF1BR28P237	Georgia Guria 7	09662	Guria, Georgia	Anakliuri OP; market^im^	2010	Georgian 1
HF1BR30P34	Georgia Samegrelo 2	09659	Samegrelo, Georgia	Dedopolis titi OP; private garden^im^	2010	Georgian 2
HF1BR30P37	Georgia Samegrelo 3	09659	Samegrelo, Georgia	Dedopolis titi OP; private garden^im^	2010	Georgian 2
HF1BR30P71	Georgia Imereti 8	09628	Imereti, Georgia	‘Lewis’ OP; experimental garden^im^	2010	Georgian 2
HF1BR30P103	Georgia Imereti 10	09672	Imereti, Georgia	‘Ganja’ OP; experimental garden^im^	2010	Georgian 2
HF1BR30P179	Georgia Imereti 11	09667	Imereti, Georgia	‘Willamette’ OP; experimental garden^im^	2010	Georgian 2
HF1BR30P189	Georgia Adjara 7	09660	Adjara, Georgia	‘Mshavala’ OP; roadside vendor^im^	2010	Georgian 4
HF1BR30P197	Georgia Adjara 8	09660	Adjara, Georgia	‘Mshavala’ OP; roadside vendor^im^	2010	Georgian 2
HF1BR30P205	Georgia Kakheti 10	09642	Kakheti, Georgia	Pshauri 2 OP; private garden (or Mshavala OP roadside)^im^	2010	Georgian 4
HF1BR32P27	Georgia Imereti 12	09627	Imereti, Georgia	KTN 30b OP; experimental garden^im^	2010	Georgian 2
HF1BR32P31	Georgia Imereti 13	09627	Imereti, Georgia	KTN 30b OP; experimental garden^im^	2010	Georgian 2
HF1BR32P49	NA	09627	Imereti, Georgia	KTN 30b OP; experimental garden^m^	2010	NA
HF1BR32P74	Georgia Kakheti 11	09641	Kakheti, Georgia	‘Nemsa’ OP; roadside vendor^im^	2010	Black Sea
HF1BR32P99	Georgia Adjara 9	09612	Adjara, Georgia	‘Gulshishvela’ OP; roadside vendor^im^	2010	Georgian 3
HF1BR32P103	Georgia Adjara 11	09612	Adjara, Georgia	‘Gulshishvela’ OP; roadside vendor^im^	2010	Georgian 3
HF1BR32P112	Georgia Adjara 13	09612	Adjara, Georgia	‘Gulshishvela’ OP; roadside vendor^im^	2010	Georgian 3
HF1BR32P116	Georgia Adjara 14	09612	Adjara, Georgia	‘Gulshishvela’ OP; roadside vendor^im^	2010	Georgian 3
HF1BR32P119	Georgia Adjara 15	09612	Adjara, Georgia	‘Gulshishvela’ OP; roadside vendor^im^	2010	Georgian 4
HF1BR32P120	Georgia Adjara 16	09612	Adjara, Georgia	‘Gulshishvela’ OP; roadside vendor^im^	2010	Georgian 3
HF1BR32P128	Georgia Adjara 17	09612	Adjara, Georgia	‘Gulshishvela’ OP; roadside vendor^im^	2010	Georgian 2
HF1BR32P135	Georgia Guria 8	09615	Guria, Georgia	‘Skheniskbili’ OP; market^im^	2010	Georgian 4
HF1BR32P148	Georgia Imereti 14	09625	Imereti, Georgia	‘Nottingham’ OP; private garden^im^	2010	Black Sea, Admixed
HF1BR32P159	Georgia Kakheti 12	09661	Kakheti, Georgia	Gulshishvela OP; private garden^im^	2010	Georgian 3
HF1BR32P166	Georgia Kakheti 14	09661	Kakheti, Georgia	‘Gulshishvela’ OP; private garden^im^	2010	Georgian 3
HF1BR32P168	Georgia Kakheti 15	09661	Kakheti, Georgia	‘Gulshishvela’ OP; private garden^im^	2010	Georgian 3
HF1BR32P174	NA	09661	Kakheti, Georgia	‘Gulshishvela’ OP; private garden^m^	2010	NA
HF1BR32P175	NA	09661	Kakheti, Georgia	‘Gulshishvela’ OP; private garden^m^	2010	NA
HF1BR32P217	Georgia Imereti 16	09666	Imereti, Georgia	KX29 OP; market^im^	2010	Georgian 2

Seedlot origins including source information, year planted, genetic group as resolved from genetic diversity studies, and previous references when available are included.

^a^ Accession code also references the original field location (Rutgers research farm, field, row number, and tree number) where the trees were established.

^b^ Other name/aliases refers to truncated reference code names used in genetic diversity studies by Muehlbauer et al. (2014) and Lombardoni et al. (2024).

^c^ Maternal parent (when available) of seedlot, and location of collection.

^d^ Genetic group or clade as resolved by Muehlbauer et al. (2014) and/or Lombardoni et al. (2024).

^e^ Accessions not resistant to eastern filbert blight but showing high tolerance, rating 3 on 0-5 scale described in Pinkerton et al. (1992).

^f^ Molnar et al. (2007).

^g^ Capik et al. (2013).

^h^ Muehlbauer et al. (2014).

^i^ Lombardoni et al. (2024).

^j^ Honig et al. (2019).

^k^ Şekerli et al., 2021.

^l^ Mehlenbacher et al. (2023).

^m^ Leadbetter et al. (2015). NA, Not Applicable.

A majority of the seedlots have been described previously including the identification of EFB resistant seedling and are referenced in **Table 1**. However, some more recent seedlots were not included in published studies, and were documented internally only as part of the Rutgers breeding program evaluations. Therefore, their collection origins are described here and are included in **Table 1** as a point of record.

Turkey: In August 2004, S. Mehlenbacher collected OP seeds from 50 different trees growing in the *Corylus* gene bank at the Horticultural Research Institute in Giresun, Turkey. The gene bank consisted of trees established by Engin Cetiner, who amassed clones of cultivars, selections, landraces, and wild trees originating from Turkey (Öztürk et al., 2017). Çalıskan and Çetiner (1992) described each tree in the collection, and their publication was used to select the trees from which to harvest seed. While most seed were utilized at OSU, a subset of each of the 50 seed lots was sent to Rutgers, from which 509 trees were planted in the field in 2006.

Latvia and Lithuania: In September 2005, T. Molnar and D. Zaurov collected five seed lots from Latvia and six from Lithuania. In Latvia, four of the seed lots were purchased from the Central Market in Riga with no information on their parentage other than assurance by the seller that they were locally sourced nuts. The fifth seed lot was a mixture of OP seeds obtained from hazelnut research plots located at the Latvia Institute of Fruit Growing in Dobele, Latvia. They were collected from seedlings originating from breeding work of Dr. Siimon, an early breeder at the institute. The seed lots from Lithuania were harvested from six different trees growing at the Lithuanian Research Center of Agriculture and Forestry in Babtai, Lithuania. In total, 306 trees from Latvia and 40 from Lithuania were planted in the field in 2006.

Estonia: In September 2005, Molnar and Zaurov also visited parts of Estonia, but seeds were not available that season. However, colleagues K. Kask and T. Paal remained in contact and in 2007, OP seeds were collected. Four seedlots, three from garden plots in Tartu, Estonia, and one from the Polli Horticultural Research Centre (Karksi-Nuia, Viljandi District, Estonia) (Kask, 2001), were shipped to New Jersey. They were germinated and yielded 203 trees that were field planted in 2008.

Russia: In September 2008, D. Zaurov collected wild *C. avellana* seeds from plants growing in a garden plot as well as wild trees in a roadside forest location north of Moscow, Russia. These seedlots (n=7) yielded 61 total trees that were planted at Rutgers in 2009. Zaurov also obtained one seedlot in 2009 from southern Russia with an unrecorded origin, from which two seedlings were planted in 2009.

### Exposure to EFB

All trees were exposed to *A. anomala* by natural spread from infected trees in adjacent breeding plots. They were also field inoculated in their 2nd and 3rd seasons after planting to promote more uniform exposure. This was accomplished by tying locally sourced diseased stems exhibiting fully formed stromata cut into 10-15 cm segments into the upper canopy of each tree before bud break in the spring (Molnar et al., 2007; Capik et al., 2013; Leadbetter et al., 2016). Several seed lots were also subject to greenhouse inoculation of *A. anomala* prior to field planting as described in Capik et al. (2013) and Leadbetter et al. (2015). This process was done to expedite exposure. It involves growing the trees in a high humidity chamber in the greenhouse and spraying the trees three times over 10 days with ascospores of *A. anomala* at a concentration of 1 x 10^6^ spores/ml. It is important to note that due to the pathogen’s latent period, symptoms of EFB are not expressed for 16-18 months following exposure. Trees inoculated in the greenhouse were planted in the field the same season as the exposure and symptoms assessed the following year. Over the course of this study, trees were subject to continual high disease pressure from infected individuals within and surrounding the populations of study (a vast majority of seedlings in these plantings were highly susceptible and EFB occurred within the plantings at devastating levels as shown in the disease ratings described in Capik et al., 2013).

### Evaluation of disease response

Each tree was visually examined for EFB disease incidence with presence or absence of visible cankers recorded, starting at 5-6 years from planting. They were also given a qualitative disease rating using a scale from 0-5 adapted from Pinkerton et al. (1992), in which a score of 0= no EFB cankers present, 1 = 1 canker present withing the entire tree, 2= multiple cankers present but only on a single limb, 3= multiple limbs containing cankers across the tree canopy, 4= a majority (>50%) of limbs containing cankers, and 5= all limbs excluding basal sprouts have cankers present and/or presence of dead limbs within the canopy from EFB. Original reports of these evaluations and identification of resistant trees are found in Molnar et al. (2007, 2018), Capik et al. (2013), and Leadbetter et al. (2015, 2016). Results of the seedlings originating from Turkey, Latvia, Lithuania, Estonia, and Russia (Moscow and southern) were not previously published although they were evaluated following the same parameters. In 2012, 29 seedlings from Turkey, 13 from Latvia, and 2 from Lithuania were classified as resistant or highly tolerant (EFB rating 0-3) to EFB. They were recovered from 23 seedlots from Turkey, 3 from Latvia, and 2 from Lithuania (T. Molnar, unpublished).

Disease evaluations were repeated in winter of 2016/2017 on all trees remaining in the foreign germplasm collection. It is important to note that this was the winter prior to the observations of most Gasaway-protected trees succumbing to EFB, which was widely apparent in the winter of 2017/2018 across the Rutgers collections. The 2016/2017 results showed only a small reduction in resistant and highly tolerant individuals from the original reports with most of the trees that exhibited new EFB infections (n=62) still displaying a high level of tolerance to the disease (EFB rating of 1, 2, or 3 based on the Pinkerton et al. (1992) scale) (Capik and Molnar, 2017). Additionally, the number of resistant or tolerant trees from unpublished seedlots dropped to 15 total (9 from Turkey, 5 from Latvia, and 1from Lithuania), representing 11 remaining seedlots (Molnar et al., 2018). New trees from Estonia (n=3) and Moscow (n=2) were also found to be resistant to EFB and originated from three and one seedlot, respectively. To update and summarize the results of the collection in one comprehensive document, a final EFB response assessment was made in January 2024 on all remaining trees – data which are reported here.

## Results and discussion

### Selections remaining resistant

**Figure 2:**
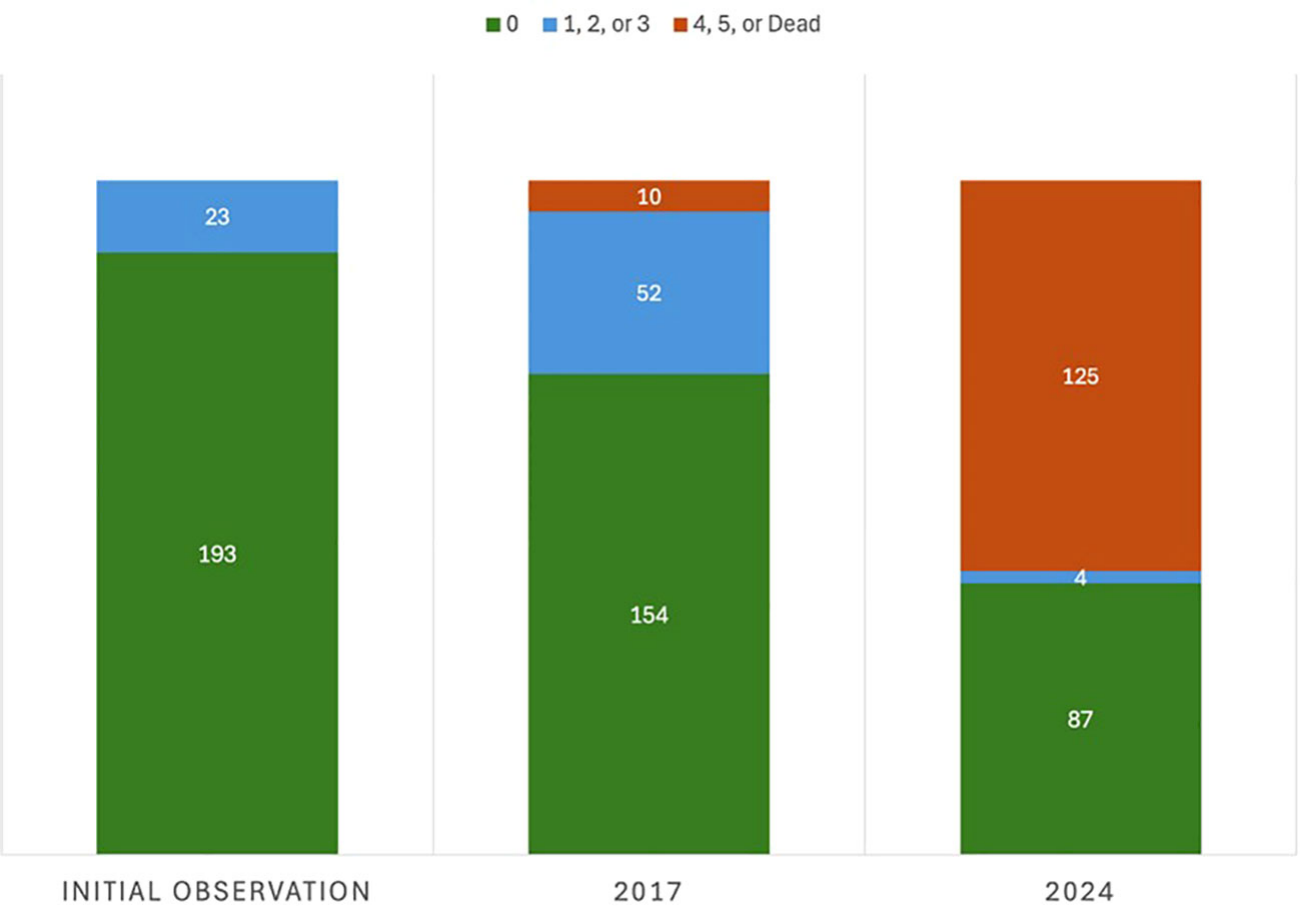
Change in disease response from 2012 to 2024 of the 216 foreign breeding selections originally deemed resistant or highly tolerant to eastern filbert blight (EFB) from the 5,226 seedling trees evaluated. Trees were scored on the 0-5 EFB rating scale developed by Pinkerton et al. (1992) where 0 = no EFB and 5 = all stems have cankers. Trees scored as 1, 2, or 3 exhibit high tolerance. Trees that died from EFB are grouped with those rating 4 and 5.

In 2024, after more than a decade of evaluation under high disease pressure, 91 foreign germplasm selections remain resistant or highly tolerant to EFB. These surviving accessions represent 1.7% of the original collection of 5,226 trees, and a 58% reduction from the 216 trees in total originally reported as resistant in Molnar et al. (2007, 2018), Capik et al. (2013), and Leadbetter et al. (2015) (**Figure 2**). Since 2017, the EFB response for the 71 newly infected individuals changed from EFB-free or highly tolerant to severe (EFB rating 4 or 5), resulting in canopy collapse and tree death. This drastic change in response suggests, when considering past performance under heavy presence of EFB, a scenario of *R*-gene breakdown. This new disease expression was identified in once resistant/highly tolerant seedlings that spanned all locations of origin; however, certain seedlots and origins suffered higher incidence and patterns in this disease expression change were evident as discussed subsequently. Fortunately, in January 2024, 56 seedlots of the original 75 continue to hold resistant individuals, and these seedlots spanned all countries of origin except for Lithuania and Moldova (**Figure 1**) These remaining individuals represent 14 of the 18 total *C. avellana* genetic clades collectively resolved by Muehlbauer et al. (2014) and Lombardoni et al. (2024) and represent inclusion in the major centers of diversity of *C. avellana*, including the Black Sea region of the Republic of Georgia (4 distinct subgroups) and Turkey, southern Europe (Spain/Italy), and the Moscow region. Consequently, the diverse backgrounds of these EFB resistant selections offer value in maintaining high genetic diversity when breeding this heterozygous, outcrossing species.

Of the Russia and Crimea selections collected in 2003 and 2005 and previously deemed resistant to EFB in Molnar et al. (2007) and Capik et al. (2013), 26 of 74 (35%) trees remain free of EFB in 2024 (**Table 2**). An additional tree, CRXR16P57 (Nikita Botanical Garden 1 #1), expressed high tolerance (rating 3) and continues to abate disease spread under high pressure. Eighteen of 25 Russian and Crimean seedlots are still represented by resistant trees and span most of the geographic regions from which seed was collected. Two additional trees from Moscow and southern Russia were collected in 2007 and 2009, respectively, and they also remain free of EFB in 2024.

Interestingly, within this material trends were present in the observed erosion of resistance that supports pathogenic variation as a potential driver for the change in disease response. New EFB expression was seen primarily in seedlots RUS-9, RUS-13, and RUS-26. Interestingly, these seedlots hold the selections H3R04P23, H3R04P28, H3R04P30, H3R07P07, H3R07P11 and H3R13P40, whose *R*-genes were recently mapped to LG6 (Komaei Koma et al., 2021; Mehlenbacher et al., 2023) and have responded similarly to the formerly resistant ‘Gasaway’ related cultivars in plots at Rutgers. This finding also suggests that seedlings of a common seedlot (generally seeds were sourced from the same mother tree) were likely carrying a shared *R*-gene. Note, in further support of resistance breakdown, Leadbetter et al. (2016) describes the EFB response of progeny of then resistant H3R04P23, H3R04P28, and H3R04P30 crossed with known EFB susceptible *C. avellana* which segregated in a pattern reflective of the presence of a dominant *R*-gene; a ratio of one resistant seedling to one susceptible seedling. However, the offspring expressing resistance in this 2016 study have since succumbed to severe EFB (T. Molnar, unpublished data).

The remaining original trees from Russia and Crimea are genetically diverse and represent 5 of 8 and 8 of 10 groups in population genetic studies by Muehlbauer et al. (2014) and Lombardoni et al. (2024), respectively. Among these selections remaining free of EFB, *R*-genes were mapped to LG2 (Holmskij Market 4#2 [H3R07P25]) and LG7 (Simferopol Market 2 #2, #3 [H3R12P58 and H3R12P62]) (Honig et al., 2019; Mehlenbacher et al., 2023).

Seedlings originating from Turkey and Latvia also saw a reduction from the 14 resistant trees in 2017. Five accessions, all from Giresun, remained free of EFB and two from Riga displayed stable tolerance (**Table 2**). The remaining resistant accessions represent four seedlots, down from six in 2017, and two distinct genetic groups (Lombardoni et al., 2024).

In 2015, 79 trees from the Republic of Georgia were found to be resistant to EFB (Leadbetter et al., 2015). By 2017, the number of resistant accessions was reduced to 49 individuals (Molnar et al., 2018) and 47 in 2024 (**Tables 1**, **2**). These individuals represent all 27 seedlots from the 2009 collections and six distinct genetic groups from the analysis by Lombardoni et al. (2024).

Five trees originating from Poland, first described in Capik et al. (2013) persist in 2024 (**Table 2**), which originate from two seedlots and represent two genetic groups (Muehlbauer et al., 2014; Lombardoni et al., 2024). Lastly, three resistant trees remain from Estonia. While SSR/SNP marker diversity data is limited for these trees, they represent three distinct seedlots obtained in 2007 from a country not otherwise represented by Rutgers introduced germplasm.

### Long-term EFB response

The reduction of resistant trees (216 to 91 trees) in this collection over time is significant and highlights the importance of evaluating EFB response over multiple years and when exposed to diverse *A. anomala* populations. Our experience, which includes the observation of resistance segregation in progeny from controlled crosses followed by severe breakdown (not published), suggests escaping disease infection is not a significant factor in the erosion of resistance observed. This premise is further supported when considering the very low frequency of resistance following early screenings (~4%), along with epidemic levels of disease in the plots, and the longevity of these plantings (14-21 years). Trees that were initially resistant grew to full maturity under exposure to the pathogen before later expressing EFB symptoms supporting an apparent breakdown of the resistance originally expressed.

The foreign selections with *R*-genes mapped to LG6 (n=6) all developed severe and fatal EFB infections around the time EFB was first observed on the ‘Gasaway’-protected trees. Whereas the selections with *R*-genes mapped to LG2 and LG7 have shown no EFB. These include H3R07P25 (Holmskij Market 4 #2) whose *R*-gene was mapped to LG2 (Honig et al., 2019), and accessions H3R12P58 (Simferopol Market 2 #2) and H3R12P62 (Simferopol Market 2 #3) with *R*-genes mapped to LG7 (Mehlenbacher et al., 2023). This finding further supports a shift in disease response driven by pathogenic variation and is corroborated by the recent study of Jacobs et al. (2024) where a wide diversity of LG6 protected cultivars and selections also succumbed to EFB despite these same clones remaining free under tests in Oregon. It also is important to note the prevalence of LG6 mapped *R-*genes in a wider assortment of evaluated germplasm. Specifically, of the 34 total sources of EFB resistance whose *R*-genes have been mapped at OSU and Rutgers, 19 (56%) have been localized to LG6, suggesting a high prevalence of *R*-genes at this gene region (Mehlenbacher et al., 2023). These mapped sources are diverse, with *R*-genes from four different *Corylus* species including many *C. avellana* sources originating throughout its native range. As a result, it is likely that some of the additional breakdowns observed in the Rutgers foreign introductions are a result of unmapped LG6 *R*-genes that were similarly overcome by EFB.

This observed breakdown of resistance across a wide pool of germplasm highlights the dynamic nature of the pathogen and the importance of breeding for durable resistance. The trees remaining resistant in the collection are genetically diverse and present the opportunity to search for additional *R* genes, those possibly even on different LGs. Expression analysis of resistance/tolerance genes can add further understanding of genetic control and mechanism for resistance. Diversifying resistance sources used in orchards is expected to help reduce selective pressure for overcoming resistance by the pathogen and can be used as part of a management strategy to extend the useful life of *R* genes. Trees containing diverse and complementary *R* genes can be used in targeted crosses as part of *R* gene pyramiding schemes. It is expected that trees protected by more than one *R* gene and/or resistance mechanism will provide a more challenging scenario for pathogen evolution and breakdown of resistance over time. And utilizing trees expressing QR (high levels of tolerance), several of which are included in this collection, can further support the development of cultivars expressing durable resistance expected to be more long lived than single *R* genes alone (Pilet-Nayel et al., 2017).

### Utilizing the EFB resistant and tolerant germplasm

Ninety-one resistant trees from 56 foreign seedlots remain at Rutgers and span much of the documented diversity in *C. avellana* (Muehlbauer et al., 2014; Lombardoni et al., 2024). Details of their origins have been assembled, and each individual tree is now included in a formalized dataset described in this manuscript (**Table 2**) to support preservation at Rutgers, external sharing including through the USDA-ARS, and continued characterization for use in breeding and additional research. Note that while strict quarantine regulations prohibit the movement of clonal *Corylus* plant material from North America to Europe and other parts of the world due to the threat of *A. anomala*, this pathogen is not seed or pollen borne thus opportunity exists to more easily share germplasm through these pathways.

The original screening efforts spanned many different plots of land on multiple Rutgers University research farms. Efforts are underway to clonally propagate and consolidate the 91 selections into a replicated trial at Rutgers University Horticultural Research Farm 3. The consolidation and replication of this material will facilitate its continued evaluation and streamline its utilization in research. Such efforts include the development of mapping populations for *R*-gene and QTL localization and expression analysis, along with the identification of self-incompatibility (S) alleles, and documentation of bloom phenology. Additional studies in progress include phenotyping the collection for nut and kernel attributes and other important production traits as described in Mehlenbacher and Molnar (2021).

## Conclusion

More than 20 years of collection and evaluation of seed-derived *C. avellana* germplasm has yielded a large population of EFB-resistant and tolerant breeding selections that have thrived under high disease pressure in the native range of the diverse EFB pathogen. While a significant proportion of the original resistant trees succumbed to EFB, believed to be due in part to changes in the pathogen population over time, the remaining trees represent a wide swath of *C. avellana* genetic diversity that constitutes a valuable genetic base from which to support future breeding and research efforts. The presence of distinct *R*-gene loci in the small number of selections evaluated (LG2, LG6, and LG7) indicates a value in searching for additional gene regions associated with resistance within the collection. These trees also present the opportunity to preserve and bolster resistance alleles through *R* gene pyramiding, and/or by crossing with trees expressing QR, to support the development of cultivars expressing durable resistance (Pilet-Nayel et al., 2017). Further, as comprehensive genomic resources are now available for *Corylus* such as described in Talbot et al. (2024) and breeding tools and technologies like genome editing, genomic selection, high throughput phenotyping, etc. are becoming more accessible to specialty crop breeding programs, this diverse collection presents a valuable resource to explore traits including disease resistance mechanisms and beyond. On a practical sense, utilizing a wide diversity of resistance sources in future orchards is suggested as an approach to reduce selection pressure on the fungus, which has been shown to overcome plant resistance both in its native range, and recently in the Willamette Valley of Oregon, where severe EFB infections on ‘Gasaway’ *R*-gene cultivars are now, for the first time, being reported on several farms (Wiman, 2023).

Currently, *A. anomala* remains isolated to North America, where regionally adapted resistant cultivars and specific management practices support hazelnut production. Beyond the confines of North America, this body of *C. avellana* germplasm represents a potentially invaluable resource if *A. anomala* is introduced to Europe or elsewhere globally, as these resistance sources are a diverse representation of the species and may be directly adapted to the regions where they originated and/or could serve as locally adapted breeding parents. By formally documenting this collection, we hope to increase awareness of this accumulated germplasm resource that is available to aid efforts in managing EFB now and in the future.

## Data Availability

The raw data supporting the conclusions of this article will be made available by the authors, without undue reservation.
